# Evaluating the Influence of *CHI3L1* and *PI3* Methylation in Allergic and Nonallergic Asthma

**DOI:** 10.3390/biom15101363

**Published:** 2025-09-25

**Authors:** Selene Baos, Lucía Cremades-Jimeno, María Ángeles de Pedro, María López-Ramos, Rubén Fernández-Santamaría, Cristina Rosales-Ariza, Joaquín Quiralte, Fernando Florido, Nicolás González-Mangado, María Jesús Rodríguez-Nieto, Germán Peces-Barba, Joaquín Sastre, Blanca Cárdaba

**Affiliations:** 1 Immunology Department, IIS-Fundación Jiménez Díaz-UAM, 28040 Madrid, Spain; selenebmuniz@gmail.com (S.B.); lucia.cremades@quironsalud.es (L.C.-J.); mpedrm1@gmail.com (M.Á.d.P.); mlr3041996@gmail.com (M.L.-R.); ruben.fsantamaria@quironsalud.es (R.F.-S.); cristina.rosales@quironsalud.es (C.R.-A.); 2 Allergy Department, Vírgen del Rocío University Hospital, 41013 Seville, Spain; joaquinquiralte@gmail.com; 3 Allergy Department, San Cecilio University Hospital, 18007 Granada, Spain; jfernandoflorido@gmail.com; 4 Pulmonology Department, University Hospital Fundación Jiménez Díaz, 28040 Madrid, Spain; ngonzalez@fjd.es (N.G.-M.); mjrodriguezn@fjd.es (M.J.R.-N.); gpeces@fjd.es (G.P.-B.); 5 Ciber de Enfermedades Respiratorias (CIBERES), 28029 Madrid, Spain; jsastre@fjd.es; 6 Allergy Department, University Hospital Fundación Jiménez Díaz, 28040 Madrid, Spain

**Keywords:** asthma, biomarkers, *chitinase-3-like 1*, DNA methylation, elafin, epigenetics, gene expression, *PI3*, YKL-40

## Abstract

Previously, we defined *CHI3L1* and *PI3* as genes related with asthma and severity by analysis of differential gene expression. In this study, we investigated the role of DNA methylation in their regulation, and their relationship with protein levels and clinical parameters. Peripheral blood mononuclear cells (PBMCs) and sera were collected from healthy controls (HCs), nonallergic asthmatic (NA), and allergic asthmatic (AA) patients. RNA and DNA were extracted from PBMCs using the trizol method. Gene expression was assessed by qRT-PCR, and DNA methylation of CpG sites near the promoters was analyzed using sodium bisulfite treatment followed by PCR amplification. DNA methylation analysis was performed using the Sequenom EpiTYPER platform. Protein levels were quantified by ELISA, and statistical analyses were carried out using GraphPad software. Consistent with previous findings, *CHI3L1* and *PI3* gene expression were significantly lower in asthmatic patients compared to controls. Conversely, CHI3L1 protein levels were higher in both patient groups, while PI3 protein showed no significant changes. DNA methylation analysis revealed higher overall DNA methylation percentages in NA and AA patients for both genes compared to HCs. Despite this, no significant correlations were observed between DNA methylation and gene or protein expression, although some correlations were observed with clinical parameters. In conclusion, *CHI3L1* and *PI3* represent potential asthma biomarkers, whose regulation may be partially influenced by DNA methylation, a mechanism more pronounced in asthmatic patients than in healthy subjects.

## 1. Introduction

Asthma is a common chronic respiratory disease, defined by the Global Initiative for Asthma (GINA) as a “heterogeneous disease, typically characterized by chronic airway inflammation” [1]. Although current therapeutic approaches are effective for many asthmatic patients, there remains an unmet need for treatment options, particularly for uncontrolled severe asthma. This is likely due to the different underlying disease mechanisms, which require further investigation. Despite its clinical heterogeneity, allergy has been implicated in 50–80% of asthmatic patients, especially in those with severe asthma [2,3]. In this context, the type 2 asthma endotype (characterized by a T2 immune response and allergic inflammation) has garnered attention in the search for biomarkers and the development of new therapeutic approaches, mainly through biological treatments targeting eosinophilic inflammation [4,5,6]. However, the non-type-2 asthma endotype, which shows a prevalence of neutrophilic or mixed neutrophilic/eosinophilic inflammation, is poorly characterized [7]. As a result, few biologically targeted therapies have been developed for this group of patients. Consequently, a key goal in asthma treatment is to develop disease-modifying therapies, which could be achieved through the development of treatments targeting specific inflammatory pathways in asthma pathogenesis. This underscores the importance of identifying and accurately characterizing patients who are more likely to respond to specific therapies, which is the goal of precision medicine [6,8,9].

Classic genetic and genomic studies have identified candidate genes of biological importance in asthma, although these genes explain only a modest proportion of the disease’s phenotypic variation. Epigenetic mechanisms could explain the environmental origins of asthma and its phenotypic variability [10,11,12,13]. A range of environmental risk factors has been proposed to contribute to the development of asthma, including allergen exposure, tobacco smoke, and air pollution [14]. The effects of these environmental factors appear to be partly mediated through epigenetic mechanisms that modify the expression of relevant genes, potentially altering molecular mechanisms involved in both health and disease. Epigenetics is defined by three criteria [15]: (i) a change in gene activity not involving a mutation, (ii) a change initiated by a signal, and (iii) an inheritable change by mitosis or meiosis that does not require the initial signal to trigger the change.

Several common epigenetic mechanisms have been identified, including DNA methylation, histone modification, chromatin remodeling, and small noncoding RNAs (21–26 nucleotides long). In asthma, various studies have demonstrated how these mechanisms can influence different aspects of the disease [16,17]. DNA methylation, especially in CpG sites near the promoter region, is associated with transcriptional repression, while hypomethylation typically results in gene overexpression [18,19]. Epigenetic mechanisms are essential for the plasticity of the cellular response provoked by environmental exposure and for the development of different cellular processes. To the extent that environmental and genetic factors are essential to the pathogenesis of allergic diseases, epigenetics likely contribute to the origins of the diseases and their phenotypic variability [20].

A major limitation of current studies on asthma epigenetics is that epigenetic modifications are often studied in heterogeneous asthma phenotypes (e.g., allergic and nonallergic asthma together), making it difficult to distinguish epigenetic modifications associated with allergic asthma from those mediating nonallergic asthma [21].

In this context, our group tries to define new and complementary molecular biomarkers able to discriminate allergic (T2-high) and nonallergic asthma (T2-low or non-T2) disease and predict disease severity in non-invasive samples. Firstly, we identified novel genes and proteins as biomarkers able to discriminate healthy control subjects (HCs) from nonallergic asthmatic patients (T2-low) and asthma severities [22]. The relevance of these biomarkers in asthma and allergic diseases was previously discussed [23,24]. Later, we defined at the genetic and protein level the ability of those biomarkers to discriminate allergic (T2-high) and nonallergic asthma (T2-low or non-T2) diseases and severity. The results showed panels of genes and proteins able to discriminate T2-high and T2-low asthma and severity [25] by easy techniques and with very good discriminatory parameters. Two of the main biomarkers defined were chitinase-3-like protein 1 (*CHI3L1*) and proteinase inhibitor 3 (*PI3*) genes that were highly decreased in PBMCs of asthmatic patients (both allergic and nonallergic) compared with healthy subjects [23,24].

*CHI3L1* is a gene that codifies a nonenzymatic chitinase-like also named YKL-40 in humans for the tyrosine (Y), lysine (K), and leucine (L) residues present in the N-terminal of the secreted form [26]. CHI3L1 has been implicated in many disorders due mainly to its broad spectrum of biological activities, as has recently been reviewed [27]. Its implication in inflammatory conditions [28,29,30,31,32,33,34,35,36], including respiratory diseases such as asthma, chronic obstructive pulmonary disease (COPD), or fibrosis has been described, but their role is not fully understood [37,38,39,40]. Several studies describe the *CHI3L1* gene or YKL-40 protein expression associated with asthma, pointing to their role as potential biomarkers to asthma [41]. However, several controversies need to be solved (i.e., their contribution as asthma biomarker, or their specificity to an asthma endotype: T2 eosinophilic asthma, non-T2 eosinophilic asthma, or both).

In addition, the *PI3* gene, located on chromosome 20q12-q13 in humans, encodes trappin-2, a 9.9kDa protein, precursor of the elafin, a low molecular weight peptide (6kDa) originally identified as a protease inhibitor in the lung and skin [42,43] that specifically inhibits neutrophil elastase (NE) and proteinase 3 [44]. Elafin functions as an antimicrobial peptide against Gram-positive and Gram-negative bacteria, and fungal pathogens and their anti-inflammatory effect in respiratory disorders has also been described [45]. However, few works have studied the relationship between *PI3* or trappin-2/elafin and asthma. In 2016, a significant inverse association between elafin levels and asthma was demonstrated, pointing to a protective role of elafin [46]. In concordance with those results, our group found a statistically significant decrease in *PI3* gene expression in PBMCs from asthmatic patients (both allergic and nonallergic) compared with HCs [23,24].

In this study, we aim to further validate the utility of CHI3L1 and PI3 expression as asthma biomarkers and explore the role of DNA methylation as an epigenetic modulator involved in their regulation. Our goal is to evaluate their potential as diagnostic biomarkers or indicators of disease severity.

## 2. Materials and Methods

### 2.1. Study Design and Subjects

The study population consisted of 54 unrelated subjects: 12 healthy control (HC) subjects, 22 nonallergic asthmatic (NA) patients, and 20 allergic asthmatic (AA) patients (allergic to airborne allergens). The NA and AA patients were diagnosed at the allergy and pulmonology departments of *Fundación Jiménez Díaz* Hospital in Madrid, while the HC subjects were recruited from the allergy departments of *San Cecilio* University Hospital in Granada and Policlinic Hospital in Sevilla.

Pulmonary function tests were conducted on asthmatic patients by determining the predicted percentage of forced vital capacity (%FVC) and forced expiratory volume in 1 s (%FEV_1_). Allergy test included a skin prick test to a panel of common allergens, including dust mites (*Dermatophagoides pteronyssinus*, *Dermatophagoides farinae*, and *Lepidoglyphus destructor*), animal epithelia (cat and dog), cockroaches (*Blatella orientalis* and *Blatella germanica*), pollens (*Cypress*, *banana shadow*, olive, mixture of grasses, *Artemisia*, *Parietaria*, and *Salsola*), and fungi (*Alternaria*, *Cladosporium*, *Aspergillus*, and *Penicillium*).

NA patients were patients diagnosed with asthma but with negative skin prick test, while AA patients were asthmatic and allergic to at least one airborne allergen. In both groups, asthma severity was classified as severe, moderate, or mild according to the Spanish Guidelines for the Management of Asthma (GEMA) [47]. Samples were collected on a day when participants had not taken any medication.

The HC group included healthy subjects with no history of respiratory diseases or allergic symptoms and negative skin prick test results.

Written informed consent was obtained from all participants in accordance with the Declaration of Helsinki. Ethical approval was granted by the research ethics committee of IIS-FJD-UAM.

Peripheral blood samples were collected from each participant and processed in the Immunology Department of the Health Research Institute-*Fundación Jiménez Díaz-UAM* (IIS-FJD-UAM) in Madrid.

### 2.2. Isolation of Peripheral Blood Mononuclear Cells (PBMCs), RNA, and DNA Extraction

PBMCs were isolated from heparinized peripheral blood samples by gradient centrifugation using Lymphoprep (Comercial Rafer, Zaragoza, Spain) according to the manufacturer’s instructions. Cell isolation was performed under sterile conditions using endotoxin-free reagents. RNA and DNA were extracted from PBMCs (10^6^ cells) using the Trizol method (Invitrogen, Carlsbad, CA, USA). The quantity and purity of the samples were assessed by spectrophotometry using a Nanodrop ND-1000 spectrophotometer (Bonsái Technologies Group, Madrid, Spain).

### 2.3. Gene Selection

*CHI3L1* and *PI3* were selected based on our previous findings [23] as two of the most promising biomarkers for asthma.

### 2.4. Differential Gene Expression Analysis by RT-qPCR

Gene expression was assessed at the Scientific Park of Cantoblanco (Madrid, Spain) using quantitative real-time PCR (qRT-PCR) with the TaqMan Gene Expression System (Applied Biosystems, Foster City, CA, USA) in 384-well microfluidic cards. Briefly, 300 ng of RNA from each sample were reverse-transcribed into cDNA using the High Capacity RNA-to-cDNA Kit (Applied Biosystems, Foster City, CA, USA). Real-time PCR was performed with Taqman Gene Expression Assays and the HT7900 System (Applied Biosystems, Foster City, CA, USA), with 40 amplification cycles. Results were analyzed using SDS software 2.1 (Applied Biosystems, Foster City, CA, USA).

Gene expression of *CHI3L1* and *PI3* was measured in triplicate, with the expression of the 18S gene used as a reference. Pre-designed TaqMan Gene Expression Assays used were *CHI3L1-Hs00609691_m1*, *PI3-Hs00160066_m1,* and *18S-Hs99999901_s1*, respectively. Relative Quantification (RQ) values were calculated using the cycle threshold (Ct) method, with the gene expression represented as 2^−ΔΔCt^, where ΔΔCt = (ΔCt_clinical group_) − (ΔCt_control group_), and ΔCt = (Ct_study gene_) − (Ct_18S_).

### 2.5. Measurement of CHI3L1 and PI3 Protein Levels

Serum samples were obtained by centrifuging peripheral blood samples without anticoagulant from all participants. Serum levels of CHI3L1 and PI3 were measured using the human chitinase-3-like 1 DuoSet ELISA (Cat# DY2599) and the Human Trappin-2/Elafin DuoSet ELISA (Cat# DY1747) (R&D Systems, Minneapolis, MN, USA).

### 2.6. Epigenetic Study by DNA Methylation Analysis

Five hundred nanograms of genomic DNA extracted from PBMCs were analyzed by Making Genetics (Pamplona, Spain) using MassARRAY EpiTYPER^®^ technology. Genomic DNA was treated with sodium bisulfite using the EZ DNA Methylation-Gold™ Kit (Zymo Research, Irvine, CA, USA). The targeted regions were amplified by PCR using specific primers (Table 1).

**Figure 1:**
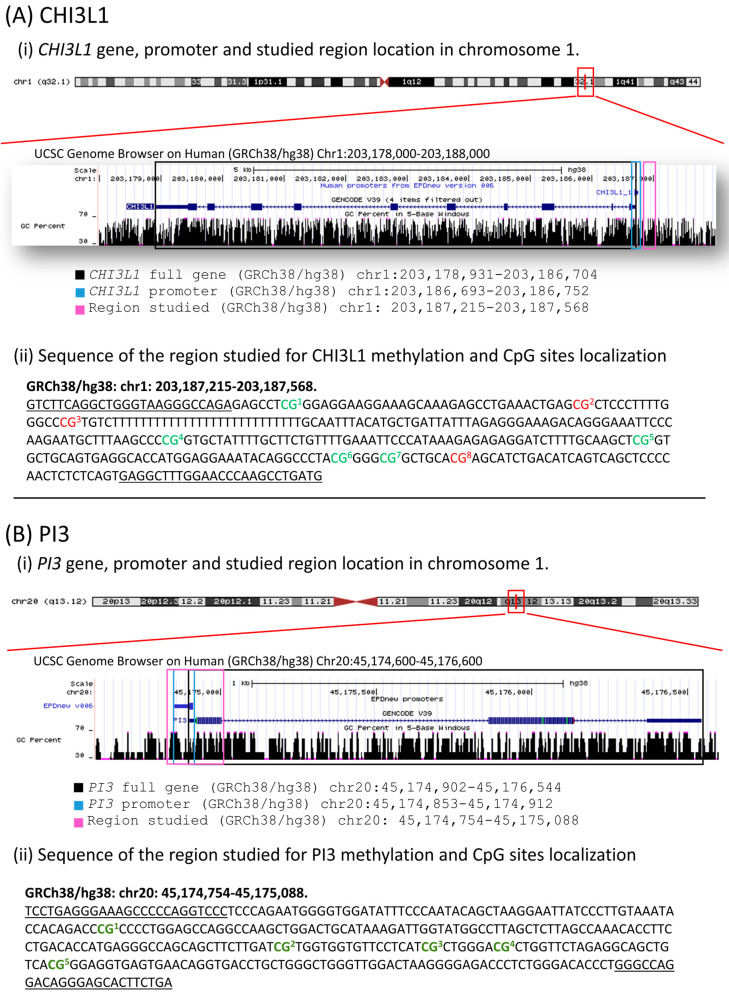
Regions studied in the DNA methylation analysis of CpG sites near to the promoter of (**A**) *CHI3L1* and (**B**) *PI3*. In green are marked the studied sites. In red are marked 3 CpG sites included in the *CHI3L1* amplified sequence but not studied.

Primers were designed with SEQUENOM^®^ EpiDesignerBETA software (http://www.epidesigner.com), including a T7 promoter sequence (reverse sequence: 5′-CAGTAATACGACTCACTATAGGGAGAAGGCT-3′) and 10 nucleotides for PCR compensation in the forward primer sequence (AGGAAGAGAG). This design amplified regions near the gene promoters, yielding an amplicon of 354 bp for *CHI3L1* and 335 bp for *PI3* (Figure 1). In total, 8 CpG sites in *CHI3L1* were analyzed, though only 5 were detected (Figure 1A); for PI3, 5 CpG sites were analyzed (Figure 1B). RNA transcription in vitro was followed by base-specific RNA cleavage in U with RNAse A. Cleavage products were analyzed using MALDI-TOF (MassARRAY^®^ Analyzer). Methylated and non-methylated cytosines were distinguished with EpiTYPER^®^ software.

### 2.7. Statistical Analysis

Statistical analyses were performed using GraphPad InStat 3.0. Differential gene expression (ΔCt values), protein expression, and DNA methylation percentages of CpG sites between clinical groups were compared using the Mann–Whitney U test.

Statistical significance was set at a two-tailed *p* value < 0.05. Correlations between DNA methylation and gene/protein expression were analyzed in each group using the non-parametric Spearman correlation test. A strong correlation was defined as r ≥ 0.80, moderate as 0.79 ≥ r ≥ 0.50, and weak as r < 0.50.

To explore the overall effect of DNA methylation across all CpG sites, multiple regression analysis was performed with DNA methylation data from all sites as independent variables and gene expression as the dependent variable. The goodness-of-fit was assessed by the R^2^ value, considering results relevant when the adjusted R^2^ closely matched the R^2^ value.

## 3. Results

### 3.1. Subjects

The studied population consisted of 12 HC and 42 asthmatic patients, 22 nonallergic (NA) patients and 20 allergic (AA) patients. The demographic and clinical characteristics of the participants are summarized in Table 2.

The NA group presented the highest mean age (58.3 ± 13.8 years), with statistically significant differences compared to the HC group (47.2 ± 9.3 years; *p* = 0.016) and the AA patients (41 ± 17.2 years; *p* = 0.0026). AA patients were the youngest, although there were no significant differences compared to the HC group. In terms of lung function, the NA group had better FVC and FEV_1_ percentages than the AA group, but without reaching statistical significance. No functional data were available for HC subjects. Regarding asthma severity, approximately half of the asthmatic patients in both groups were diagnosed with moderate or mild asthma, while the other half had severe asthma. The highest IgE levels were observed in the AA group (405.2 ± 472.7 kU/L), followed by the NA group (93.5 ± 87.5 kU/L) and the HC group (41.9 ± 75.9 kU/L). Differences were statistically significant between the clinical groups and healthy controls (HC vs. NA: *p* = 0.006; HC vs. AA: *p* < 0.0001), as well as between the two asthmatic groups (NA vs. AA: *p* = 0.0005).

### 3.2. CHI3L1 and PI3 Gene and Protein Expression, and DNA Methylation Analysis

#### 3.2.1. Gene and Protein Expression of CHI3L1 and PI3

**Figure 2:**
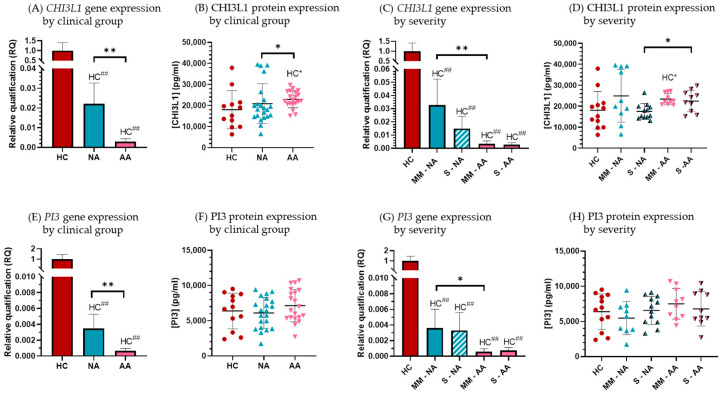
CHI3L1 (**A**–**D**) and PI3 (**E**–**H**) gene and protein expression according to clinical group and severity subgroup in Healthy Control subjects (HC), Nonallergic Asthma (NA), and Allergic Asthma (AA) patients. Asthma severity is indicated as S for severe and MM for moderate–mild diagnosis. The gene expression graphs represent the mean gene levels and the standard error, measured by RT-qPCR in 12 HC, 20 NA, and 20 AA, expressed as the relative quantification (RQ) for the clinical groups compared to the HC group (see Section 2). The protein expression is shown as the mean levels ± standard deviation (SD) of CHI3L1 and PI3 in serum, quantified by ELISA in all subjects included in the study. Statistical analysis was performed using the nonparametric Mann–Whitney test using the Graph-Pad InStat 3.0 program. Statistically significant differences between HC and the indicated disease group are shown as HC* (*p* < 0.05) and HC^##^ (*p* < 0.0001). Statistically significant differences among clinical groups are shown as * (*p* < 0.05) and ** (*p* < 0.01).

*CHI3L1* and *PI3* showed a significantly lower gene expression in the asthmatic patients’ groups compared to HC, with allergic patients showing the lowest expression (Figure 2). Interestingly, the protein expression results were discordant: AA patients exhibited the highest CHI3L1 protein expression, while PI3 serum levels were similar across all clinical groups (Figure 2).

Regarding gene expression, relative quantification (RQ) values for *CHI3L1* were 0.02 ± 0.01 in the NA group and 0.003 ± 0.001 in the AA group, significantly lower than in HC subjects, with significant decreases for both clinical groups (*p* < 0.0001). Additionally, there were statistically significant differences between the NA and AA groups (*p* = 0.0016) (Figure 2A). At the protein level, the mean serum CHI3L1 protein levels in HC were 18,074.5 ± 8986.5 pg/mL, with slightly higher expression in NA (20,910.9 ± 9435.6 pg/mL) and AA (22,958.2 ± 3999.5 pg/mL). These differences were statistically significant when comparing the AA group to HC (*p* = 0.02) and to the NA group (*p* = 0.03) (Figure 2B).

For the *PI3* gene expression (Figure 2E), the RQ values compared to HC were 0.003 ± 0.001 and 0.0006 ± 0.0003 in the NA and AA group, respectively, with significant decreases (*p* < 0.0001 for both comparisons). *PI3* gene expression was significantly lower in AA patients compared to NA patients (*p* = 0.007). Serum PI3 protein levels showed large variations, and the differences between clinical groups did not reach statistical significance (Figure 2F).

When gene and protein expression were analyzed by asthma severity (patients with severe asthma vs. moderate or mild asthma), we observed that *CHI3L1* gene expression was lower in severe patients compared to moderate–mild patients in both NA and AA groups. In the NA group, severe patients showed *CHI3L1* gene expression 100 times lower than HC subjects (RQ = 0.01 ± 0.008; *p* < 0.0001), while in the AA group, severe patients had *CHI3L1* gene expression 500 times lower (RQ = 0.002 ± 0.001; *p* < 0.0001). In the moderate–mild patients, a significant decrease in *CHI3L1* expression compared to HC was also observed, with a greater reduction in AA patients (Figure 2C).

At the protein level, lower serum CHI3L1 protein levels were observed in severe patients compared to moderate–mild patients, although these differences were not statistically significant. However, clinical groups showed higher protein expression than HC, in contrast to the gene expression results (Figure 2D) with statistically significant increases in moderate–mild AA patients.

For *PI3* gene expression, both severity subgroups in both asthmatic patient groups showed significantly lower levels compared to HC, with a decrease of approximately 300 times in the NA group and 1500 times in the AA group (*p* < 0.001 for both). The expression of moderate–mild AA patients was also significantly lower than NA moderate–mild patients (*p* = 0.03). (Figure 2G). No significant differences were observed in serum PI3 protein levels between severity subgroups (Figure 2H).

#### 3.2.2. DNA Methylation Analysis of CHI3L1 and PI3

**Figure 3:**
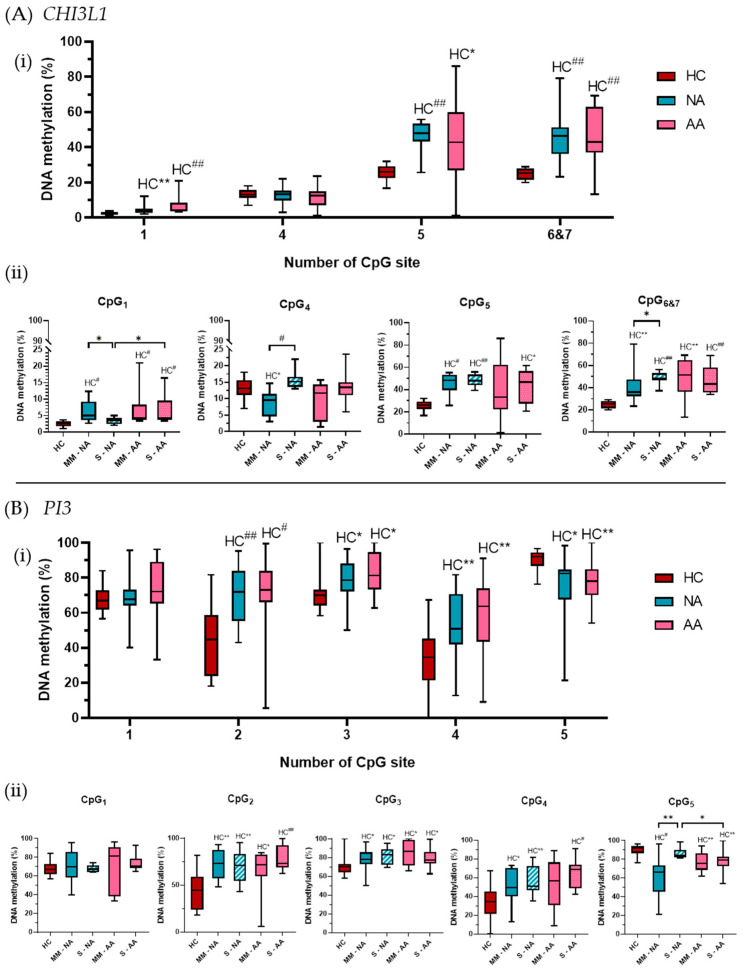
(**A**) CHI3L1 and (**B**) PI3 methylation percentages of the CpG sites studied according to clinical groups (**i**) and severity of the disease (**ii**) in the first population studied (P1). HC: healthy control, NA: nonallergic asthma, AA: allergic asthma, MM: moderate or mild diagnosis, S: severe diagnosis. The DNA methylation percentage in the CpG sites studied was measured in all the subjects from the studied population, and the results are shown in box and whisker graphs, where the median, standard deviation, maximum, and minimum values are represented. These data were compared among groups and severities by Nonparametric Mann–Whitney test using the Graph-Pad InStat 3 program. Statistically significant differences between HC and the indicated disease group are shown as HC* (*p* < 0.05), HC** (*p* < 0.01), HC^#^ (*p* < 0.001), and HC^##^ (*p* < 0.0001). Statistically significant differences among indicated clinical groups are shown as * (*p* < 0.05) and ** (*p* < 0.01).

In order to better understand the possible regulatory mechanisms implicated in these gene expression differences between groups, we studied the DNA methylation, an epigenetic regulatory mechanism, analyzing CpG sites near to the promoter of the genes. In the DNA methylation analysis of *CHI3L1* and *PI3*, we observed differences in DNA methylation percentages between the clinical groups and HC subjects. Figure 3 summarizes the *CHI3L1* (A) and *PI3* (B) methylation results, in both clinical groups (i) and according to asthma severity (ii).

For *CHI3L1*, five CpG sites near the gene promoter were studied. The results showed that CpG_1_ and CpG_4_ had lower DNA methylation percentages than CpG_5_ and CpG_6&7_. However, all the CpG sites (except CpG_4_) showed higher DNA methylation in the asthmatic groups compared to HC subjects (Figure 3A(i)).

The CpG_1_ site had the lowest DNA methylation levels across all groups, with mean values less than 10%, especially in HC subjects (2 ± 1%). The clinical groups showed significantly higher DNA methylation, with 5 ± 3% in NA (*p* = 0.0023) and 7 ± 5% in AA (*p* < 0.0001).

When patients were analyzed by asthma severity, both AA severity subgroups maintained the significant difference with HC subjects, while only moderate–mild NA patients showed significant differences (*p* < 0.0001 for all comparisons). Severe NA patients exhibited DNA methylation levels (3 ± 1%) similar to HC subjects, significantly lower than the moderate–mild NA group (6 ± 3%, *p* = 0.011) and severe AA patients (7 ± 5%, *p* = 0.04) (Figure 3A(ii)).

CpG_4_ showed similar DNA methylation percentages across HC subjects (13 ± 3%) and the asthmatic groups (13 ± 5% in NA and 11 ± 5% in AA) (Figure 3A(i)). Regarding asthma severity, severe patients had higher DNA methylation than moderate–mild patients, in both asthma groups, but the differences were statistically significant only in the NA group (*p* = 0.0003). Moreover, moderate–mild NA patients showed significantly lower DNA methylation compared to HC subjects (*p* = 0.011) (Figure 3A(ii)).

The other three sites (CpG_5_ and CpG_6&7_) showed higher DNA methylation percentages in all groups, around 50% in asthmatic patients. The CpG_5_ site had the highest DNA methylation in HC and NA groups, while AA patients exhibited higher DNA methylation in CpG_6&7_. In both sites, HC subjects had significantly lower DNA methylation percentages than the asthmatic patients. For CpG_5_, HC subjects had 26 ± 4% DNA methylation, while the clinical groups had nearly 50% of DNA methylation: 47 ± 8% in NA (*p* < 0.0001 vs. HC) and 42 ± 21% in AA (*p* = 0.011 vs. HC) (Figure 3A(i)). These differences were maintained in NA, regardless of severity (45 ± 10%, *p* = 0.0003 in mild–moderate NA; and 48 ± 5%, *p* < 0.0001 in severe NA), but only severe AA patients showed significantly higher DNA methylation (44 ± 15%) than HC subjects (26 ± 4%, *p* = 0.010), while moderate–mild AA patients had a similar DNA methylation percentage (40 ± 27%) (Figure 3A(ii)). Finally, DNA methylation in the CpG_6&7_ sites was higher in the clinical groups, with mean percentages of 45 ± 12% in NA and 48 ± 16% in AA, compared to 25 ± 3% in HC subjects. These differences were significant (*p* < 0.0001) (Figure 3A(i)). When analyzed by severity, these differences remained significant for both groups: severe NA had 49 ± 5%, and moderate–mild NA had 41 ± 16% DNA methylation (*p* < 0.0001 and *p* = 0.0026 vs. HC, respectively), while severe AA had 47 ± 13%, and moderate–mild AA patients had 49 ± 19% DNA methylation (*p* < 0.0001 and *p* = 0.0046 vs. HC, respectively). Statistically significant differences between the two severity subgroups in NA patients were observed (*p* = 0.017) (Figure 3A(ii)).

For *PI3*, all CpG sites showed high DNA methylation levels, with significant differences between HC subjects and asthmatic patients in four of them (Figure 3B(i)), three with a lower methylation in HC subjects (CpG_2_ to CpG_4_) and one showing higher methylation in HC (CpG_5_). Nonetheless, the first CpG site showed similar mean methylation levels in all groups, without differences between them, neither globally nor by severity (Figure 3B(i,ii)).

In the CpG_2_ site, we found lower DNA methylation in the HC subjects (43 *±* 20%) than in the clinical groups (70 ± 16% and 73 ± 20% in NA and AA, respectively). These differences with the HC subjects were statistically significant in both groups: NA (*p* = 0.0006) and AA (*p* = 0.0001) (Figure 3B(i)). Comparing severities, we observed similar DNA methylation percentages between moderate–mild and severe patients in both asthma groups. Again, the DNA methylation in all the subgroups was statistically higher than that observed in the HC subjects (Figure 3B(ii)). Similar results were obtained for the CpG_3_ site, where the mean DNA methylation in HC subjects (71 ± 11%) was statistically lower than that observed in NA patients (80 ± 10%; *p* = 0.011) and AA patients (82 ± 12%; *p* = 0.006) (Figure 3B(i)). Again, those differences were maintained independently of the severity of the asthmatic individuals (Figure 3B(ii)). In the CpG_4_ site, again, the HC subjects presented statistically significant lower DNA methylation percentages (33 ± 20%) than those observed in both asthmatic groups: NA (54 ± 17%; *p* = 0.0028) and AA (58 ± 22%; *p* = 0.0027) (Figure 3B(i)). When asthma groups were separated by severity, both NA subgroups maintained the statistical significance with the HC group (57 *±* 15%, *p* = 0.004 in severe NA; 51 *±* 19%, *p* = 0.03 in moderate–mild NA), while only the severe AA subgroup (64 *±* 15%, *p* = 0.0006) had a significantly higher percentage of DNA methylation than the HC subjects (Figure 3B(ii)).

Opposite results were observed in the CpG_5_ site, where mean DNA methylation was higher in the HC subjects (90 ± 7%) than in the clinical groups: NA (75 ± 19%; *p* = 0.0085) and AA (77 ± 11%; *p* = 0.0013) (Figure 3B(i)). Analyzed by severity, we observed significant differences between the DNA methylation in HC and both severity subgroups in AA patients (78 ± 12%, *p* = 0.011 and 77 ± 10%, *p* = 0.004 in severe and moderate–mild patients, respectively), while with moderate–mild NA patients it was only 62 ± 22%, *p* = 0.0024. We also found statistically significant differences between different severities within the NA group (*p* = 0.0051) and between both severe asthma subgroups (*p* = 0.009) (Figure 3B(ii)).

#### 3.2.3. Correlation of CHI3L1 and PI3 Expression with DNA Methylation and Clinical Parameters

To understand the potential regulatory mechanisms behind the observed gene expression differences, we performed a correlation analysis between gene expression and each CpG site separately. In both genes we observed, in general, higher DNA methylation percentages in the asthmatic groups compared with the HC, in accordance with the lower gene expression observed in the clinical groups. However, these correlations were not statistically significant, in either the clinical groups or severity subgroups.

A multiple regression analysis was performed to assess the influence of global DNA methylation on gene expression, using the DNA methylation data from all the sites studied as independent variables and gene expression as dependent variables. These data showed that the global DNA methylation of *CHI3L1* in moderate–mild AA patients was highly related with their gene expression (R^2^ = 0.82, adjusted R^2^ = 0.61). No significant correlations were found for *PI3*.

Finally, with the aim to better understand the possible implications of those parameters in the clinical manifestations of the disease, we analyzed the correlation between biomarker expression and clinical characteristics of the patients in each group. For CHI3L1, significant correlations were found in the NA group. A weak negative correlation was observed between FEV_1_ and the *CHI3L1* ΔCt (Rs = −0.45; *p* = 0.04) and CHI3L1 serum levels (Rs = 0.46; *p* = 0.03), indicating that patients with higher ΔCt (thus lower relative quantification) or lower protein expression had worse lung function. When patients were segregated by severity, FVC and CHI3L1 serum levels showed a strong negative correlation in severe NA (Rs = −0.78; *p* = 0.003) and moderate–mild NA patients (Rs = −0.82; *p* = 0.008). In moderate–mild NA patients, there was a significant negative correlation between FEV_1_ and *CHI3L1* ΔCt (Rs = −0.76; *p* = 0.02).

In contrast, PI3 serum protein levels showed only a significant negative correlation with FVC (Rs = −0.48; *p* = 0.03), with no significant correlations found for *PI3* gene expression or within severity subgroups.

## 4. Discussion

In this study, we analyzed the gene and protein expression of CHI3L1 and PI3 in peripheral samples (PBMCs and serum) to evaluate their potential as biomarkers for different clinical asthma phenotypes (allergic asthma, AA, and nonallergic asthma, NA) and according to disease severity, building on our previous work [22,23,24,25]. Additionally, we investigated DNA methylation in promoter regions of these genes to explore its possible role in gene regulation.

### 4.1. CHI3L1: Expression, Protein Levels, and DNA Methylation

CHI3L1 (chitinase-3-like protein 1, also known as YKL-40) is a non-enzymatic chitinase-like protein produced by various cell types, including macrophages, neutrophils, fibroblasts, endothelial cells, and cancer cells [48,49,50,51,52,53]. It exhibits a broad range of biological activities relevant to inflammatory processes and tissue remodeling, such as promoting cell proliferation, survival, immune cell activation and differentiation, and regulation of extracellular matrix metabolism [27].

Although its role in respiratory diseases like asthma, COPD, and fibrosis has been reported, its precise function remains incompletely understood. In COPD, elevated serum levels have been associated with tissue inflammation and remodeling [37,38]. In animal models and patients with lung injury and fibrosis, diverse roles have been described, including a potentially protective effect in hyperoxia acute lung injury in mice [39] and increased levels in cystic fibrosis patients [40]. Regarding asthma, CHI3L1 plays a key role in CD4+ T cell polarization and Th2 inflammation. It is expressed in activated T cells and Th2 cells, regulating Th1/Th2 differentiation [54]. Murine models show increased CHI3L1 expression during Th2-driven inflammatory responses induced by allergens such as ovalbumin and dust mites, with reduced Th2 inflammation observed in *CHI3L1* knockout mice [55]. CHI3L1 also promotes bronchial smooth muscle cell proliferation via MAPK and NF-κB pathways [27,56].

However, results are conflicting as to whether CHI3L1 serves as a specific biomarker for type 2 (T2) asthma. Some studies link it to IL-13-dependent T2 inflammation [55,57,58], while others suggest it is not T2-specific and may be elevated in non-eosinophilic asthma [59,60,61]. Similarly, associations between CHI3L1 polymorphisms, circulating YKL-40 levels, and asthma features vary across studies, possibly due to population genetic differences [62,63,64,65,66,67,68]. A recent study indicated that genetic variations in *CHI3L1* modulate circulating YKL-40 via DNA methylation profiles but showed no direct association with childhood asthma [69].

In our cohort, comprising healthy controls and patients with AA and NA, we confirmed a significant decrease in *CHI3L1* gene expression in PBMCs from asthma patients compared to controls (Figure 2A), consistent with prior findings [22,23,24,25]. Conversely, serum CHI3L1 protein levels were elevated in all asthmatic groups, especially in AA patients with mild-to-moderate disease, supporting its potential role as a T2-specific biomarker driven by IL-13 [55,57,58].

Regarding associations with lung function, significant correlations between CHI3L1 gene and protein expression and FEV_1_ were observed only in NA patients, with additional correlations with FVC when stratifying by severity. These findings suggest a possible role for CHI3L1 in the nonallergic asthma phenotype and align with previous reports linking circulating YKL-40 levels with lung function [62,70,71].

To explain differences in gene expression, we analyzed the DNA methylation as a mechanism that could be implicated in their regulation, since the epigenetic regulation of *CHI3L1* has been linked to other diseases [72,73]. Here the DNA methylation of eight CpG sites in the *CHI3L1* promoter was studied. CpG sites 5 and 6 and 7 exhibited the highest methylation levels, roughly double in asthma patients compared to controls, with statistically significant differences in both phenotypes, regardless of severity (Figure 3A). This hypermethylation may partly explain the reduced gene expression observed, although individual correlations were not significant. Multiple regression analysis combining all CpG sites showed a strong association with gene expression (R^2^ = 0.82), specifically in mild-to-moderate AA patients, suggesting an accumulative epigenetic regulatory effect.

In summary, despite sample size limitations, our data indicate a potential involvement of DNA methylation in regulating *CHI3L1* expression in asthma, though other regulatory mechanisms likely contribute. A bioinformatic analysis using UCSC Genome Browser on Human (GRCh38/hg38) searching for Transcription factors and SNPs in the *CHI3L1* region studied showed predicted binding sites (Table S1) for 4 Zinc finger proteins (*ZNF263*, *ZNF574, ZNF701,* and *ZNF531* or *ZFP14*) in the CpG1 site of *CHI3L1,* remarking *ZNF263* that has been associated with regulation in asthma [74,75]). Also, in the *CHI3L1* amplicon studied, we found a match with *IRF5* (interferon regulatory factor 5) and *HES6* (hes family bHLH transcription factor 6) in the two CpGs (two and three, respectively) that could not be detected by technical reasons in this study. Both, especially *IRF5*, have been extensively related to asthma regulation [76,77,78,79,80].

### 4.2. PI3: Expression, Protein Levels, and DNA Methylation

Regarding *PI3*, which encodes trappin-2 (precursor of elafin), an antimicrobial peptide and protease inhibitor with anti-inflammatory effects [42,43,44,45,46], its role in asthma is less explored. Consistent with previous reports [23,24,25,46], we observed a significant reduction in *PI3* gene expression in PBMCs of asthma patients, particularly in AA, while serum protein levels showed no significant differences but a slight tendency to increase in AA (Figure 2B). Correlations with lung function parameters were weak, limited to a negative correlation between serum PI3 and FVC.

DNA methylation analysis of five CpG sites in PI3 showed generally higher DNA methylation in asthmatic patients compared to healthy controls (Figure 3B) but no significant correlation with gene expression, either individually or combined, suggesting that DNA methylation is unlikely the primary regulator of *PI3* in PBMCs. As for *CHI3L1*, we search for Transcription factors and SNPs in the *PI3* region studied. The *PI3* amplicon analyses also showed predicted binding sites for five transcription factors, four in CpG_1_— *KLF16* (KLF transcription factor 16), *SP3* (Sp3 transcription factor), *KLF15* (KLF transcription factor 15), and *SP9* (Sp9 transcription factor)—and one, *FERD3L* (Fer3 like bHLH transcription factor), in the CpG_5_ site. Many of them have been associated with asthma regulation, mainly *SP3* [81,82,83] and *KLF15* [84,85,86,87]. Interestingly, we found two SNPs in CpG_3_ and CpG_4_ sites (Table S2).

This information should be analyzed in depth by functional studies to understand their potential implication.

Limitations include the use of PBMCs, a heterogeneous cell population with variable epigenetic patterns and cell composition changes associated with asthma that may affect DNA methylation analyses [88]. Grouping mild and moderate patients may also obscure clear conclusions. Additionally, clinical stability, medication, and other factors (smoking, obesity, environmental exposure) were not accounted for and should be addressed in future studies. A longitudinal design with detailed clinical and environmental data and larger cohorts would better clarify the role of these biomarkers.

In conclusion, our results support *CHI3L1* and *PI3* as gene biomarkers for allergic and nonallergic asthma. However, the impact of DNA methylation on their expression remains unclear. To our knowledge, this is the first study examining epigenetic DNA modifications of *PI3* in asthma. Further studies in larger, well-controlled populations are necessary to elucidate the significance of these epigenetic mechanisms in respiratory diseases.

## 5. Conclusions

Our findings support previous evidence indicating altered expression of CHI3L1 and PI3 in asthma, particularly at the gene level. While DNA methylation differences were observed, especially for *CHI3L1*, their impact on gene regulation remains inconclusive. To our knowledge, this is the first study to examine *PI3* methylation in the context of asthma, and although results are preliminary, they highlight the need for further research into epigenetic regulation in asthma pathogenesis.

## Figures and Tables

**Table 1:** CHI3L1 and PI3 primers used for the quantitative analysis of DNA methylation, in regions near to the promoter of the genes.

Gene	Primers (5′–3′)	Amplicon (Base Pairs)	CpG Sites—Coverage
*CHI3L1*	F-aggaagagagGTTTTTAGGTTGGGTAAGGGTTAGA R-cagtaatacgactcactatagggagaaggctCATCAAACTTAAATTCCAAAACCTC	354	8–5
*PI3*	F-aggaagagagTTTTGAGGGAAAGTTTTTAGGTTTT R-cagtaatacgactcactatagggagaaggctTCAAAAATACTCCCTATCCTAACCC	335	5–5

**Table 2:** Demographic and clinical characteristics of the study population.

		HC Subjects	NA Patients	AA Patients
N		12	22	20
Gender, N (%)	Male	4 (33.3)	6 (27.3)	4 (20)
	Female	8 (66.7)	16 (72.7)	16 (80)
Age (years), mean ± SD		48.5 ±9.5	58.3 ±13.8 *	41 ±17.2 ^#^
Asthma severity, N (%)	Moderate/mild	-	10 (45.5)	10 (50)
	Severe	-	12 (54.5)	10 (50)
Pulmonary function, mean ± SD	FVC (%)	-	80.6 ±24.4	72.4 ±16.5
	FEV1 (%)	-	75.6 ±23	73.1 ±16.1
Blood analysis, mean ± SD	Total IgE (kU/L)	41.9 ± 75.9	93.5 ± 87.5 **	405.2 ± 472.7 ***^,##^
	Eosinophils (cells/mm^3^)	-	278.6 ± 162.5	480.1 ± 288.8

%FVC: percentage of forced vital capacity; %FEV1: percentage of forced expiratory volume in 1 s. *, **, *** Statistically significant comparisons (*p* < 0.05, *p* < 0.01, and *p* < 0.0001, respectively) between the indicated group and the C group. ^#^, ^##^ Statistically significant comparisons (*p* < 0.01 and *p* < 0.001, respectively) between the indicated group and the NA group.

## Data Availability

The original contributions presented in this study are included in the article/Supplementary Materials. Further inquiries can be directed to the corresponding author.

## Supplementary Materials

The following supporting information can be downloaded at: https://www.mdpi.com/article/10.3390/biom15101363/s1, Table S1: SNPs and transcription factors associated with the CpG sites near the *CHI3L1* gene promoter; Table S2: SNPs and transcription factors associated with the CpG sites studied near the *PI3* gene promoter.

## Abbreviations

The following abbreviations are used in this manuscript:

AA	Allergic Asthmatic
FEV_1_	Forced expiratory volume in 1 s
FVC	Forced vital capacity
GEMA	Spanish Guidelines for the Management of Asthma
GINA	Global Initiative for Asthma
HC	Healthy control subject
NA	Nonallergic Asthmatic
PBMCs	Peripheral Blood Mononuclear Cells
T2	Type 2
