# Supplementary material for: Evaluating the Influence of CHI3L1 and PI3 Methylation in Allergic and Nonallergic Asthma
Source: Biomolecules. 2025 Sep 25;15(10):1363. doi: 10.3390/biom15101363 (PMC12564470; doi:10.3390/biom15101363)
Supplement: Supplementary file 1 [file biomolecules-15-01363-s001.zip › biomolecules-3837737-supplementary.pdf]

Supplementary

Evaluating the Influence of CHI3L1 and PI3 Methylation in Allergic and Nonallergic Asthma

Selene Baos [1,†], Lucía Cremades-Jimeno [1,†], María Ángeles de Pedro [1], María López-Ramos [1], Rubén Fernández-Santamaría [1], Cristina Rosales-Ariza [1], Joaquín Quiralte [2], Fernando Florido [3], Nicolás González-Mangado [4,5], María Jesús Rodríguez-Nieto [4,5], Germán Peces-Barba [4,5], Joaquín Sastre [5,6] and Blanca Cárdaba [1,6,*]

1 Immunology Department, IIS-Fundación Jiménez Díaz-UAM, 28040 Madrid, Spain; selenebmuniz@gmail.com (S.B.); lucia.cremades@quironsalud.es (L.C.-J.); mpedrm1@gmail.com (M.Á.d.P.); mlr3041996@gmail.com (M.L.-R.); ruben.fsantamaria@quironsalud.es (R.F.-S.); cristina.rosales@quironsalud.es (C.R.-A.)

2 Allergy Department, Vírgen del Rocío University Hospital, 41013 Seville, Spain; joaquinquiralte@gmail.com

3 Allergy Department, San Cecilio University Hospital,18007 Granada, Spain; jfernandoflorido@gmail.com

4 Pulmonology Department, University Hospital Fundación Jiménez Díaz, 28040 Madrid, Spain; ngonzalez@fjd.es (N.G.-M.); mjrodriguezn@fjd.es (M.J.R.-N.); gpeces@fjd.es (G.P.-B.)

5 Ciber de Enfermedades Respiratorias (CIBERES), 28029 Madrid, Spain; jsastre@fjd.es

6 Allergy Department, University Hospital Fundación Jiménez Díaz, 28040 Madrid, Spain

* Correspondence: bcardaba@fjd.es

† These authors contributed equally to this work.

Table S1. SNPs and transcription factors associated with the CpG sites studied near the *CHI3L1* gene promoter.

CpG site	CpG site location	SNP	SNP location	Transcription Factor (TF)	TF binding site (TFBS) - predicted
CpG_1	chr1: 203,187,246-203,187,247	-	-	ZNF263	chr1:203,187,247-203,187,253
				ZNF574	chr1:203,187,233-203,187,246
				ZNF701	chr1:203,187,238-203,187,254
				ZFP14	chr1:203,187,239-203,187,253
CpG_2	chr1: 203,187,280-203,187,281	-	-	IRF5	chr1:203,187,269-203,187,282
CpG_3	chr1: 203,187,296-203,187,297	-	-	HES6	chr1:203,187,292-203,187,301
CpG_4	chr1: 203,187,392-203,187,393	-	-	-	-
CpG_5	chr1: 203,187,451-203,187,452	-	-	-	-
$CpG_{6\&7}$	chr1: 203,187,492-203,187,493; chr1: 203,187,497-203,187,498	-	-	-	-
CpG_8	chr1: 203,187,505-203,187,506	-	-	-	-

Table S2. SNPs and transcription factors associated with the CpG sites studied near the *PI3* gene promoter.

CpG site	CpG site location	SNP	SNP location	Transcription Factor (TF)	TF binding site (TFBS) - predicted
CpG_1	chr20: 45,174,844-45,174,845	-	-	KLF16	chr20:45,174,840-45,174,850
				SP3	chr20:45,174,840-45,174,850
				KLF15	chr20:45,174,841-45,174,848
				SP9	chr20:45,174,841-45,174,850
CpG_2	chr20: 45,174,946-45,174,947	-	-	-	-
CpG_3	chr20: 45,174,964-45,174,965	rs41282752 G/A	chr20:45,174,965	-	-
CpG_4	chr20: 45,174,972-45,174,973	rs17333103 C/T	chr20:45,174,972	-	-
CpG_5	chr20: 45,174,996-45,174,997	-	-	FERD3L	chr20:45,174,983-45,174,996
